# Physicochemical Design and *In Vitro* Evaluation of Diterpene Phytol-Loaded Solid Lipid Nanoparticles

**DOI:** 10.1021/acsabm.5c00940

**Published:** 2025-10-31

**Authors:** Lannya C. S. Tavares-Pessoa, Alaine M. dos Santos-Silva, Anne Emmanuelle C. S. Melo, Bolívar P. G. de Lima Damasceno, Matheus F. Fernandes-Pedrosa, Alianda M. Cornélio, Arnobio Antônio da Silva-Júnior

**Affiliations:** † Laboratory of Pharmaceutical Technology and Biotechnology − TecBioFar, Graduate Program in Pharmaceutical Sciences, Department of Pharmacy, 28123 Federal University of Rio Grande do Norte (UFRN), Natal-RN 59012-570, Brazil; ‡ Graduation Program in Pharmaceutical Sciences, Center for Biological and Health Sciences, State University of Paraíba (UEPB), Campina Grande-PB 58429-500, Brazil; § Department of Morphology, Federal University of Rio Grande do Norte (UFRN), Natal-RN 59078-970, Brazil

**Keywords:** Colloidal dispersions, solid lipid nanoparticles, natural compounds, slow drug release, nanonutraceuticals

## Abstract

Phytol (PHY) is a water immiscible natural diterpene with interesting pharmacologic and antioxidant properties with great potential for application in foods, cosmetics, and pharmaceuticals. In this letter, a natural 1,3-distearyl-2-oleyl glycerol (TG1) extracted from *Platonia insignis* Mart was explored as a lipid matrix for preparing uniformly sized and stable PHY-loaded solid lipid nanoparticles (SLN). Different tested compositions induced spherical (<300 nm), stable SLN with high encapsulation efficiency (>90%), able to induce slow release and a dose-dependent cytotoxic effect of PHY. These achievements corroborated with a promising nanocarrier for PHY and are able to be tested in different application fields.

## Graphical abstract

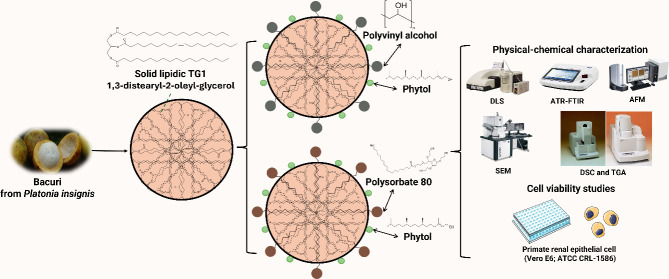

Phytol (3,7,11,15-tetramethylhexadec-2-en-1-ol) (PHY) is a naturally occurring compound as a product of chlorophyll metabolism, which can be found in the green leaves of various medicinal plants, including *Cannabis sativa* L. This diterpene (C20H40O) belongs to a wide group of unsaturated long chain and immiscible branched acyclic alcohols, which has several reported pharmacological properties, including a neuroprotective effect.^1,2^ Solid lipid nanoparticles (SLN) are a promising nanocarrier to obtain aqueous colloidal dispersions of essential oils and immiscible compounds in water, such as diterpenes.^3,4^ Nonpolar compounds can be dissolved in organic solvents containing specific solid lipids, forming nanodroplets in water, and stabilized by surfactants. In this context, it is interesting to use a natural lipid, such as 1,3-distearyl-2-oleyl glycerol (TG1), which can be extracted from *Platonia insignis* Mart. seed, popularly known as “bacurizeiro”, found in north and northeast of Brazil.^5,6^

Colloidal nanocarriers based on natural lipids, such as TG1, a natural lipid extracted from *Platonia insignis* Mart*.*, offer important advantages, such as for example, a antioxidant effect. In this study, different surfactants and surfactant/lipid ratio compositions were explored using emulsification with the solvent evaporation method to induce self-assembled aqueous nanodispersions containing PHY. All formulations were prepared under the same experimental conditions, and details of the preparation and characterization are provided in the Supporting Information. Initial results shown in Table 1 tested a fixed TG1 concentration in the organic phase (0.5%w/v) stabilized with polysorbate 80 (P80) in an aqueous phase (0.1–0.5% w/v), suggesting the highest surfactant concentration to be used for PHY nanoencapsulation. The same was used for the reaction with vinyl alcohol (PVA). Parameters as mean diameter, PDI, and EE% were considered suitable for evaluation of the physical stability of SLN.^7–11^

**Table 1:** Design of Different Solid Lipid Nanoparticles (SLN) Formulations^a^

	Composition (mg/mL of SLN)								
Samples	TG1	PVA	P80	PHY	Hydrodynamic diameter ± SD (nm)	PdI ± SD	ZP ± SD (mV)	DL (%)	EE (%)
SLN-80	3.0	–	1.4	–	*	*	*	–	–
SLN-P80	3.0	–	2.8	–	119.4 ± 0.45	0.38 ± 0.01	–36.02 ± 0.04	–	–
SLN-P80	3.0	–	4.2	–	144.7 ± 13.5	0.48 ± 0.02	–25.2 ± 1.03	–	–
SLN-P80	3.0	–	5.6	–	189.7 ± 24.9	0.29 ± 0.11	–28.5 ± 0.08	–	–
SLN-P80	3.0	–	7.0	–	150.0 ± 0.99	0.31 ± 0.03	–25.0 ± 0.09	–	–
SLN-PVA	3.0	7.0	–	–	265.3 ± 0.63	0.15 ± 0.03	–24.9 ± 1.23	–	–
PHY-SLN-P80	3.0	–	7.0	0.3	171.45 ± 2.6	0.36 ± 0.01	–25.1 ± 0.06	2.77	92.4
PHY-SLN-PVA	3.0	7.0	–	0.3	292.29 ± 6.37	0.16 ± 0.03	–25.4 ± 1.01	2.68	89.2

a ∗, phase separation; PdI, polydispersity index; SD, standard deviation; DL, drug loading; EE, encapsulation efficiency. Measurements were performed 24 h after preparing nanoparticles. Experimental values are expressed as mean ± standard deviation (*n* = 3).

ATR-FTIR spectra recorded for blank SLN, PHY-SLN, and their isolate compounds (Figure 1A, B) revealed slight stretches in PVA bands identified in SLN, which increased with PHY loading. The same was identified for characteristic signs of TG1, corroborating with high PHY nanoencapsulation.^12^

**Figure 1:**
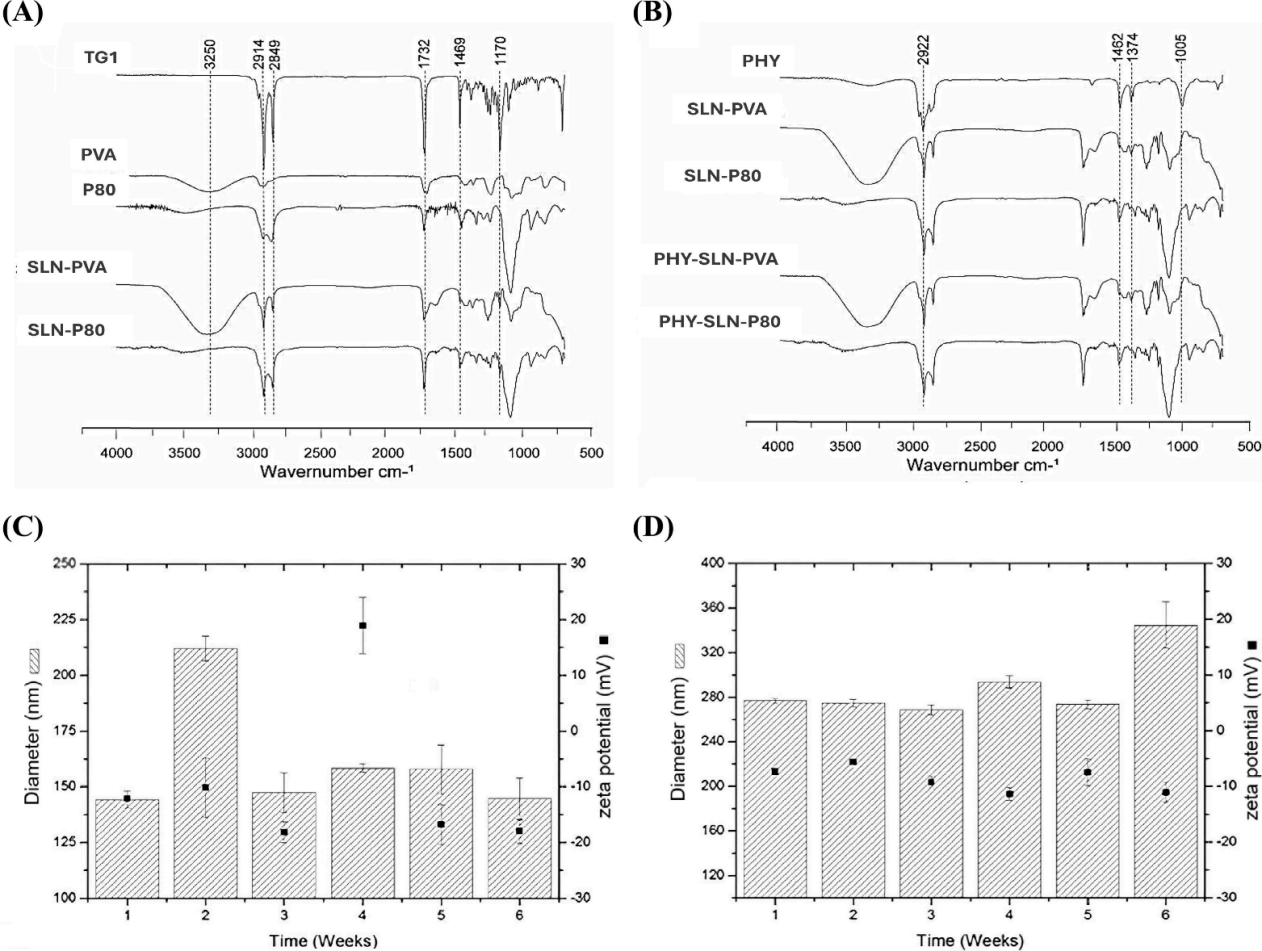
FTIR analysis of TG1, PVA, P80, SLN-PVA, and SLN-P80 (A). FTIR analysis of PHY, SLN-PVA, SLN-P80, PHY-SLN-PVA, and PHY-SLN-P80 (B). Physical stability for 6 weeks for PHY-SLN-P80 (C) and PHY-SLN-PVA (D).

The physical stability of PHY-loaded SLN was followed for 6 weeks (Figure 1C, D). No phase separation was observed for both tested samples, but the identified fluctuation in particle size and zeta potential for PHY-SLN-P80 corroborated with superior performance for PHY-SLN-PVA. Negative zeta potential values measured in the first week (Figure 1) decreased after 24 h of preparation (Table 1). Steric stabilization is considered in this study by using nonionic surfactants. Even considering Ostwald ripening phenomenon, surfactant diffusion from SLN to aqueous media is expected, especially at the first moments after preparation.^11^ PHY-SLN -PVA exhibited consistent physicochemical behavior, with no evidence of incompatibility or physical instability phenomena such as flocculation, sedimentation, creaming, or coalescence.^11,14,15^

Thermal analysis recorded for TG1, PHY, and different formulations of SLN (Figure 2A–D) showed that TG1 presents great thermal stability, with Tonset > 300 °C (red line). TGA curves (Figure 2A) for blank SLN (blue line) and PHY-loaded SLN (green line) exhibited similar behavior, suggesting an improvement of thermal stability of PHY (gray line). The same did not occur for samples stabilized with P80 (Figure 1C), in which blank SLN were superior than PHY-loaded SLN. This outcome also corroborated with superior stability of samples stabilized with PVA.^16,17^

**Figure 2:**
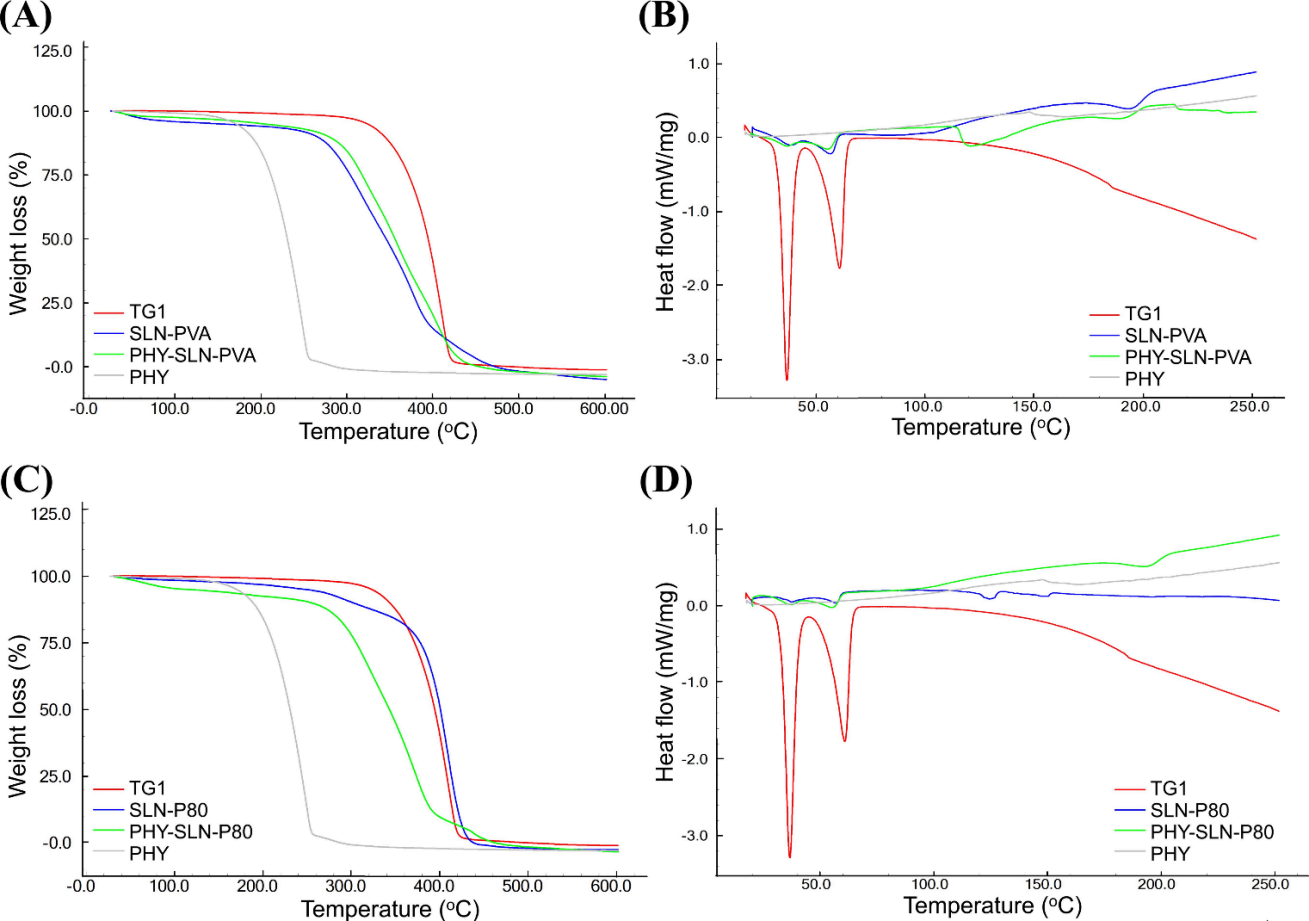
Comparisons of TGA and DSC curves for PHY-SLN-PVA (A, B) and PHY-SLN-P80 (C, D), respectively.

The DSC curve of TG1 (red line) exhibited two endothermic events, recorded at 36.7 and 60.7 °C (Figure 2B). Similar and slight signs were also detected in the blank SLN (blue line) and PHY-loaded SLN (green line), corroborated with similar solid structures for two samples stabilized with PVA. Figure 2D suggests greater amorphization in samples stabilized with P80. The achievement for TG1 is consistent with the expected melting for 1,3-distearoyl-2-oleoylglycerol and tristearin triglyceride, respectively. Polymorphic transitions and some nucleation of tristearin triglyceride with some phase separation phenomena can occur within the TG1 lipid matrix from natural sources.^17,18^ Sakellari et al. reported polymorphic lipid transitions within SLN with melting events ranging from approximately 35–45 and 55–65 °C, attributed to different compounds,^19^ reinforcing the association between these thermal events and specific compounds present in natural lipid matrices.^20^ The absence of additional melting peaks related to free PHY in the DSC curves indicates complete solubilization of PHY within the lipid matrix of the nanoparticles. Differences observed in DSC curves corroborate different encapsulation efficiencies for two surfactants.

Figure 3 illustrates *in vitro* release of phytol solution (PHY) and phytol-loaded SLN prepared with PVA and P80, as well as AFM and SEM images of the phytol-loaded SLN stabilized with PVA (PHY-SLN-PVA). Figure 3A shows that both free phytol and phytol SLN with Tween 80 (PHY-SLN-P80) exhibited a rapid release profile. In contrast, the PHY-SLN-PVA system demonstrated a prolonged release profile, with release of approximately 47% over about 600 min. Figure 3B and C shows the morphology of phytol-SLN prepared with PVA assessed by AFM and SEM images, respectively, which exhibited uniform and spherical nanoparticles, corroborating DLS measurements. In addition, the drug release curve from the nanoparticles (PHY-SLN-PVA) was subjected to four different linear diffusion kinetic models: first-order, Freundlich, Bhaskar, and parabolic diffusion models, respectively.^12,21,22^ The drug release constant (*k*) and correlation coefficient (*r*) calculated for each model are shown in Table S1 (Supporting Information).

**Figure 3:**
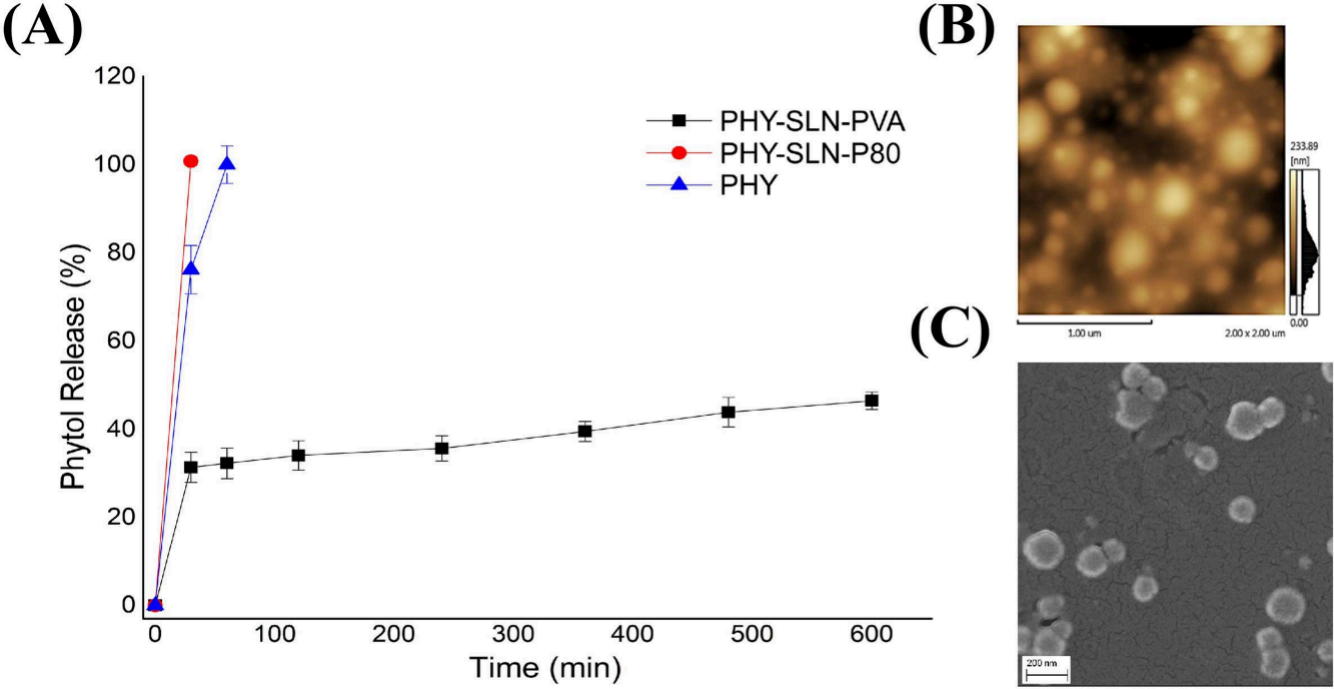
(A) Experimental *in vitro* release profile from different samples using free-PHY, PHY-SLN-PVA, and PHY-SLN-P80. (B) 2D AFM and (C) SEM image for PHY-SLN-PVA.

Figure 4 shows cell viability studies performed in Vero cells (American Type Culture Collection, ATCC: CCL-81) by using a MTT assay with two distinct blank SLN at 24 and 48 h (Figure 4A, B) suggested that SLN stabilized with P80 present greater cytotoxicity compared with PVA. Thus, PHY-loaded SLN-PVA was selected for studies at 24 and 48 h (Figure C) and comparisons with PHY at 24 and 48 h (Figure 4D). The superior cytotoxicity of P80 and how polymeric surfactants interacting with cell is established in the literature.^23–25^

**Figure 4:**
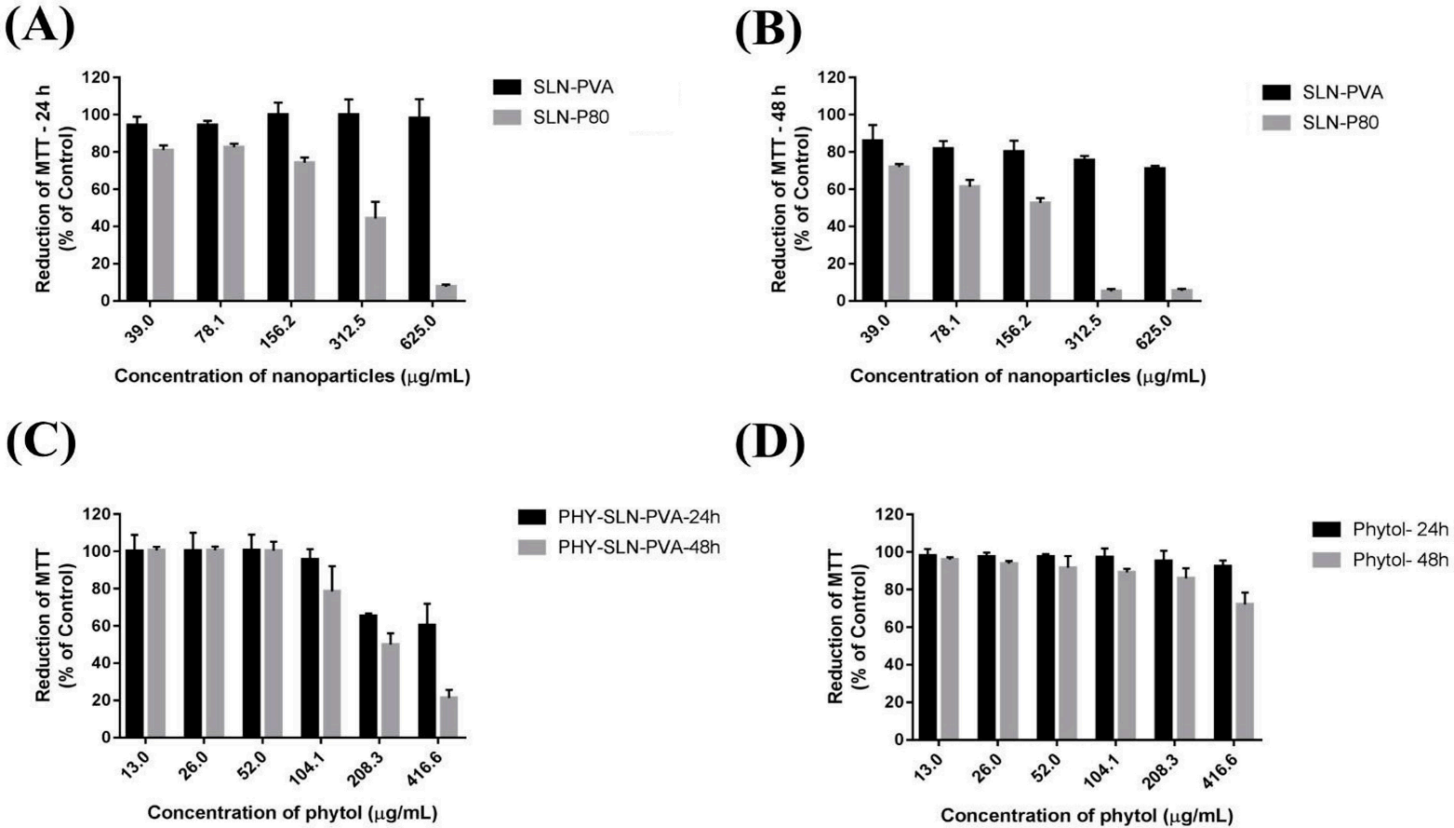
Cytotoxicity effect of and SLN and PHY-SLN-PVA as a function of the tested concentration, using the MTT assay in Vero cells incubated for 24 h (A) and 48 h (B) in relation to the concentration of SLN and (C, D) in relation to PHY concentration.

Experimental achievements highlight the potential of self-assembled PHY-loaded SLN-PVA, as spherical, stable, and uniform nanoparticles with high PHY encapsulation efficiency. Therefore, TG1-based SLN showed promising features as nanocarriers for phytol and should be further investigated to improve efficacy of their pharmacological properties.

## Supplementary Material



## Notes

Experimental methods and detailed information of preparation and characterization of solid lipid nanoparticles and results from mathematical treatment of the *in vitro* release study for the formulation PHY-SLN-PVA (Table S1) (PDF)

## Abbreviations

PHY: phytol
TG1: 1,3-distearyl-2-oleyl-glycerol
DMSO: dimethyl sulfoxide
DCM: dichloromethane
PdI: polydispersity index
SD: standard deviation
DL: drug loading
EE: encapsulation efficiency
PHY- SLN-PVA: solid lipid nanoparticle of PVA loaded with phytol.
PHY-SLN-P80: solid lipid nanoparticle of Tween-80 loaded with phytol
