# Supplementary material for: Physicochemical Design and In Vitro Evaluation of Diterpene Phytol-Loaded Solid Lipid Nanoparticles
Source: ACS Appl Bio Mater. 2025 Oct 31;8(11):9634–8. doi: 10.1021/acsabm.5c00940 (PMC12628322; doi:10.1021/acsabm.5c00940)
Supplement: Supplementary file 1 [file mt5c00940_si_001.pdf]

Supporting information

Physicochemical design and *in vitro* evaluation of diterpene phytol-loaded solid lipid nanoparticles

*Lannya C. S. Tavares-Pessoa [1], Alaine M. dos Santos-Silva [1], Anne Emmanuelle C. S. Melo [1], Bolívar P.G. de Lima Damasceno [2], Matheus F. Fernandes-Pedrosa[1], Alianda M. Cornélio[3], Arnóbio Antônio da Silva Júnior [1]**

[1] Laboratory of Pharmaceutical Technology and Biotechnology – TecBioFar, Graduate Program in Pharmaceutical Sciences, Department of Pharmacy, Federal University of Rio Grande do Norte (UFRN), Natal – RN, 59012-570, Brazil.

[2] Graduation Program in Pharmaceutical Sciences, Center for Biological and Health Sciences, State University of Paraíba (UEPB), Campina Grande-PB, 58429-500, Brazil.

[3] Department of Morphology, Federal University of Rio Grande do Norte (UFRN), Natal-RN, 59078-970, Brazil.

*Corresponding Author:

E-mail: arnobio.silva@ufrn.br

Preparation and Characterization of solid lipid nanoparticles

Materials

PHY diterpene was supplied by Sigma-Aldrich (St. Louis, MO, USA). The polyvinyl alcohol (PVA), having a viscosity molecular mass of 4.7 x 10^4 g/mol purchased from Vetec (São Paulo, Brazil). The 1,3-distearyl-2-oleyl-glycerol (TG1) was donated by the Federal University of Piauí (Teresina, Brazil). The use of plant material was conducted under authorization from the National System for Management of Genetic Heritage and Associated Traditional Knowledge (SISGEN) n° ACB9C9D. Polysorbate 80 (Tween® 80), Alamar blue, and dimethylsulfoxide (DMSO) were supplied by Sigma-Aldrich (St. Louis, MO, USA). Dichloromethane (DCM) and ethanol were purchased from Labsynth® (São Paulo, Brazil). The purified water (1.3 μS) was prepared from reverse osmosis purification equipment model OS50 LX (Gehaka, SP, Brazil). All the other chemicals and reagents were of analytical grade.

Preparation of solid lipid nanoparticles

Blank-solid lipid nanoparticles (SLN) were prepared using TG1 as lipid matrix. The parameters of the emulsification-solvent evaporation method were adjusted according to the composition of different formulations[1,2]. Briefly, 30 mg of TG1 dissolved in 6 mL of DCM and injected into 14mL of the aqueous phase containing the surfactant. The emulsification occurred at an output flux of 1 mL/min under magnetic stirring at 720 rpm at 25 ± 2 ºC. The emulsification followed in Ultra-Turrax® equipment (IKA T18 - Labortechnik, Staufen, Germany) for 18 minutes. The solvent evaporation was at 25 ºC, under magnetic stirring at 720 rpm overnight. The organic and aqueous phase were filtered using 0.45 μm teflon membrane (Sartorius®, São Paulo, Brazil). Samples were stored in hermetically sealed glass vials and stored at 25 ± 2 ºC. All experiments were performed in triplicate.

Formulation design of solid lipid nanoparticles

In this experiment, two types of surfactants were tested, the polymeric (PVA) and the non- ionic polysorbate 80 for producing small and uniform SLN by using the parameters described above. Initially, the concentration of TG1 was fixed at 0.5% (*w/v*) in the organic phase. The concentration of PVA and polysorbate 80 corresponded to 0.5% (*w/v*) in the aqueous phase.

Preparation of PHY-loaded solid lipid nanoparticles

Organic phase (6mL) containing 3mg of PHY and 30mg of TG1, to obtain PHY:TG1 ratio of 1:10 (*w/w*), was as injected at a constant flow rate of 1 mL/min into 14 mL of an aqueous phase containing 0.5% (w/v) of tested surfactant, ensuring proper emulsification of the system. The emulsification followed the parameters described above.

Particle size and zeta potential measurements

The particle size was assessed as mean hydrodynamic diameter and polydispersity index (PDI) were assessed by using Dynamic Light Scattered (DLS) in a particle size analyzer Zeta Size NanoZS (Malvern Instruments, Malvern, UK) at 659 nm wavelength, 173° detection angle, and at 25 °C. Zeta potential measurements were performed in the same equipment applying a field strength of about 5.9 $V \cdot cm^{-1}$ by using the electrophoretic mobility. The measurements were performed for at least ten determinations for each sample diluted at 1:100 (v/v) with purified water. All experiments were performed in triplicate, 24 hours after preparing nanoparticles, and data were expressed as mean ± standard deviation (SD) [3,4].

Atomic Force Microscopy (AFM) and Scanning Electronic microscopy (SEM)

AFM and SEM images were taken to evaluate morphologic aspects of selected PHY-loaded SLN. For AFM analyses, the colloidal dispersions were freshly diluted in

purified water with a ratio of 1:25 (v/v) and dropped in a cover slip, dried under desiccator for 24 h and then analyzed in an AFM equipment SPM-9700 (Shimadzu, Tokyo, Japan), at room temperature with a cantilever non-contact, 1 Hz scanning. For the SEM images, one drop of each sample was placed on a washed microscope carbon slide and dried under a desiccator for 24 h and then in a Field Emission Gun Scanning Electron Microscope Carl (FEG-SEM) (Zeiss, Auriga®) [5].

Encapsulation efficiency

The samples were centrifuged at 16,000 g for 60 min. at 4 ºC using the ultracentrifuge filter Vivaspin 2, Ultra-15 MWCO 100 kDa (Sartorius®, São Paulo, Brazil). Taken volume of 500 µL of supernatant was diluted with ethanol to 10mL (1:19 v/v) and analyzed at 239 nm by using UV-Vis spectrophotometry at 25ºC, using 1cm quartz cuvette (Thermo Fisher Scientific, 60S Evolution, Madison, WI, US). The drug mass in supernatant was estimated from drug concentration (µg/mL), calculated using the linear regression equation ($y = 0.0045x + 0.005$, r= 0.999), extracted from the standard curve, which was constructed using the same analytical parameters. All analyses were performed in triplicate and data expressed as mean ± standard deviation (SD). The encapsulation efficiency (EE) was calculated by using the Eq. (1) [3-6]

EE% = (total drug mass – drug mass in supernatant) / total drug mass x 100 Eq. (1)

Physicochemical stability

The free-drug SLN and PHY-loaded SLN were stored in hermetically closed flasks at 25 ± 2 °C for six weeks. The average particle size and the polydispersity index (PdI) were assessed using dynamic light scattering (DLS) at 659 nm, with a detection angle of 90° described for particle size measurements. Analysis was performed in triplicate and all data expressed as mean ± standard deviation (SD) [3-6].

Attenuated total reflectance Fourier transforms infrared spectroscopy ATR-FTIR

The PHY interactions with TG1 in the SLN were monitored using ATR-FTIR spectra[13]. Colloidal dispersions of free-drug and phtol-loaded SLN were concentrated using the vacuum concentrator Centrivap (Labconco, Kansas City, USA) for 7 hours. The isolated compounds (PHY, PVA, polysorbate 80, and TG1) were also analyzed. The spectra were recorded at 20 scans, with a resolution of 4 cm^{-1} between 4000 and 700 cm^{-1} in a ATR-FTIR spectrophotometer IR Prestige 21 (Shimadzu, Tokyo, Japan) [3-6].

Thermal analyses

Differential scanning calorimetry (DSC) analyses were performed on a DSC equipment model Q100 (TA Instruments, New Castle, USA). Samples (4 mg) in hermetically sealed platinum pans were analyzed at a rate of 10 °C/min over a temperature range of 0–250 °C. All analyses were performed in the N_2 atmosphere at 50 mL/min. Calibration was performed using the melting point of standard indium (m.p. 156.6 °C and ΔH = 28,45 J/g) [7-8]..

The thermogravimetric analysis (TGA) analyses were performed in thermogravimetric analyses (TGA) were performed on a TGA-50 thermogravimetric analyzer (Shimadzu, Tokyo, Japan). The samples of the nanosystems were weighed (5 mg) and placed in an alumina pan using the same conditions reported for DSC analyses 10 °C/min, N_2 atmosphere at 20 mL/min) over a temperature range of 0–600 °C. The first derivative analyses (DTG) of thermogravimetric curves were used to identify the Tonset of thermal events [7-8]..

In vitro PHY release study

The *in vitro* release of PHY from SLN was assessed in Franz vertical diffusion cells (Crown Scientific, Sommerville, USA), thermostatized at 37 ± 0.5 ºC[10]. In the donor compartment, 1.0 mL of distinct colloidal dispersions were applied and hermetically

sealed. The receptor compartment filled with 11.0 mL of buffer phosphate solution (KH_2PO_4, 0.05M, pH= 7.4) was separated by a synthetic cellulose acetate 0.45 µm filter (Sartoruius®, São Paulo, Brazil), which was previously hydrated with phosphate buffer for 24 h. The cells remained under magnetic stirring at 360 rpm, and aliquots of 1.0 mL were taken at specific intervals for analysis UV spectrophotometry (Thermo Fisher Scientific, 60S Evolution, Madison, WI, US) at 239 nm. The same volume of freshly buffer solution was replaced to the medium to maintain the sink conditions. All analyses were performed in triplicate and data expressed as mean ± standard deviation (SD) [7-9]..

Cell viability studies

The viability of Vero cells against PHY solution, blank SLN and PHY-SLN were evaluated using the MTT (3-methyl-[4-5-dimethylthiazol-2-yl]-2,5-diphenyltetrazolium bromide) assay[7-9]. The viable cells retain the capacity to reduce MTT because of the succinate dehydrogenase enzyme activity found in mitochondria. The cells were plated in 96 well plates at a density of $2x10^5$ cells/well and cultured with L-15 medium with 10% FBS and maintained at 37°C and 5% CO_2. After 24 h, the samples were filtered using a 0.45 µm Millipore filter and added in the plate. The concentrations of PHY. The same concentrations were used for PHY-SLN and blank SLN. After 24 and 48 hours of incubation, the supernatant was removed and 50 µL of L-15 medium without FBS (Fetal Bovine Serum) containing MTT (final concentration 5 mg/mL) was added to each well. The plate was incubated at 37 °C and 5% CO_2 for 4 hours. After this time, MTT diluted culture medium was removed and 100 µL of dimethyl sulfoxide (DMSO) were added to each well. The plates protected from light, and placed under stirring for 15 min, have the absorbance measured at 540 nm, using microplate reader (Biotek®, Epoch model).

Results of in vitro drug release

Table S1- Mathematical treatment of the *in vitro* release study for the formulation PHY-SLN-PVA.

**Kinetic Model**	**Equation**	***k***	***r***
First order	$\ln(M^t/M^\infty) = kt$	$0.0036\ h^{-1}$	0.90
Bhaskar	$\lg(M^t/M^\infty) = kt^{0.65}$	$0.3639\ h^{0.65}$	0.92
Freundlich	$(M^\infty - M^t)/M = kt^b$	69.357 h	0.96
Parabolic diffusion	$(1 - (M^t/M^\infty)/t = kt\ 0.5 + a.$	$0.1447\ h^{-0.5}$	0.97

Note: M^t/M^∞ , *t*, *k, r* are the fractional released drug, release time, drug release rate constant, and correlation coefficient, respectively. The *a* and *b* are linear constants whose chemical significance is not clearly solved.

References

(1) Lima, T. L. C.; Souza, L. B. F. C.; Tavares-Pessoa, L. C. S.; Dos Santos-Silva, A. M.; Cavalcante, R. S.; de Araújo-Júnior, R. F.; Cornélio, A. M.; Fernandes-Pedrosa, M. F.; Chaves, G. M.; da Silva-Júnior, A. A. Phytol-Loaded Solid Lipid Nanoparticles as a Novel Anticandidal Nanobiotechnological Approach. *Pharmaceutics* **2020**, *12* (9), 1–19. https://doi.org/10.3390/pharmaceutics12090871.

(2) dos Santos-Silva, A. M.; Caland, L. B.; do Nascimento, E. G.; Oliveira, A.L.C.S.L.; de Araújo-Júnior, R. F.; Cornélio, A. M.; Fernandes-Pedrosa, M. F.; da Silva-Júnior, A. A. Self-Assembled Benznidazole-Loaded Cationic Nanoparticles Containing Cholesterol/Sialic Acid: Physicochemical Properties, In Vitro Drug Release and In Vitro Anticancer Efficacy. *Int J Mol Sci* **2019**, *20* (9), 2350. https://doi.org/10.3390/ijms20092350.

(3) dos Santos-Silva, A.M.; de Caland, L.B.; de Melo Doro, P.N.; Oliveira, A.L.C.S. L.; de Araújo-Júnior, R.F.; Fernandes-Pedrosa, M.F. do Egito, E.S.T.; da Silva-Junior, A.A. Hydrophilic and hydrophobic polymeric benznidazole-loaded nanoparticles: Physicochemical properties and in vitro antitumor efficacy. *Journal of Drug Delivery Science and Technology*

2019, 51, 700-707. https://doi.org/10.1016/j.jddst.2019.04.005.

(4) dos-Santos-Silva, E.; Alves-Silva, M.F.; Medeiros, J.S.; Santos-Cavalcante, R.; Cornélio, A.M.; Fernandes-Pedrosa, M.F.; do Egito, E.S.T.; de Araújo-Júnior, R.F.; da Silva-Júnior, A.A. Colloidal properties of self-assembled cationic hyperbranched-polyethyleneimine covered poly lactide-co-glycolide nanoparticles: Exploring modified release and cell delivery of methotrexate, *Journal of Molecular Liquids* **2020,** 315, 113721, https://doi.org/10.1016/j.molliq.2020.113721.

(5) dos Santos-Silva, E.; Torres-Rêgo, M.; Gláucia-Silva, F. Carvalho Feitosa, R.; Lacerda, A.F. Rocha, H.A.O.; Fernandes-Pedrosa, M.F. da Silva-Júnior, A.A. Cationic plga nanoparticle formulations as biocompatible immunoadjuvant for serum production and immune response against bothrops jararaca venom, *Toxins* (**2022**), 14(12), 888; https://doi.org/10.3390/toxins14120888

(6) Chetoni, P.; Burgalassi, S.; Monti, D.; Tampucci, S.; Tullio, V.; Cuffini, A. M.; Muntoni, E.; Spagnolo, R.; Zara, G. P.; Cavalli, R. Solid Lipid Nanoparticles as Promising Tool for Intraocular Tobramycin Delivery: Pharmacokinetic Studies on Rabbits. *European Journal of Pharmaceutics and Biopharmaceutics* **2016**, *109*, 214–223. https://doi.org/10.1016/j.ejpb.2016.10.006.

(7) de Oliveira, E.G., de Caland, L.B., de Oliveira, A.R.; de Calannd,L.B.; Machado P.R.; Farias, K.L.; Melo, D.M.A. Costa, T.R.; Fernandes-Pedrosa, M.F.; Cornelio, A. M. ; da Silva-Júnior, A.A. Monitoring thermal, structural properties, methotrexate release and biological activity from biocompatible spray-dried microparticles. *Journal of Thermal Analysis and Calorimetry* **(2017)** 130, 1481–1490. https://doi.org/10.1007/s10973-017-6547-2.

(8) de Oliveira, A.R.; Mesquita, P.C.; Machado, P.R.L.; Farias, K.J.S.; de Almeida, Y.M.B.; Fernandes-Pedrosa, M.F.; Cornélio, A.M.; do Egito, E.S.T. da Silva-Júnior, A.A.Monitoring structural features, biocompatibility and biological efficacy of gamma-irradiated methotrexate-loaded spray-dried microparticles, *Materials Science and Engineering: C* **(2017)**, 80, 438-448, https://doi.org/10.1016/j.msec.2017.06.013.

(9) Torres-Rêgo, M.;Gláucia-Silva, F.; Soares, K.S.R.; Souza, L.B.F.C.; Damasceno, I.Z.; dos

Santos-Silva, E. Lacerda, A.F.; Chaves, G.M. da Silva-Júnior, A.A.; Fernandes-Pedrosa, M.F. Biodegradable cross-linked chitosan nanoparticles improve anti-Candida and anti-biofilm activity of TistH, a peptide identified in the venom gland of the Tityus stigmurus scorpion, *Materials Science and Engineering: C,* **(2019),** 103 https://doi.org/10.1016/j.msec.2019.109830.
